# A systematic review on the generative AI applications in human medical genetics

**DOI:** 10.3389/fgene.2025.1694070

**Published:** 2026-01-20

**Authors:** Anton Changalidis, Yury Barbitoff, Yulia Nasykhova, Andrey Glotov

**Affiliations:** Department of Genomic Medicine D.O. Ott Research Institute of Obstetrics, Gynaecology, and Reproductology, St. Petersburg, Russia

**Keywords:** diagnostics, genetic diseases, large langauge models, LLM, transformers

## Abstract

Although traditional statistical techniques and machine learning methods have contributed significantly to genetics and, in particular, inherited disease diagnosis, they often struggle with complex, high-dimensional data, a challenge now addressed by state-of-the-art deep learning models. Large language models (LLMs), based on transformer architectures, have excelled in tasks requiring contextual comprehension of unstructured medical data. This systematic review examines the role of generative Artificial Intelligence (AI) methods in human medical genomics, focusing on the genetic research and diagnostics of both rare and common diseases. Automated keyword-based search in PubMed, bioRxiv, medRxiv, and arXiv was conducted, targeting studies on LLM applications in diagnostics and education within genetics and removing irrelevant or outdated models. A total of 195 studies were analyzed, highlighting the prospects of their applications in knowledge navigation, analysis of clinical and genetic data, and interaction with patients and medical professionals. Key findings indicate that while transformer-based models perform well across a diverse range of tasks (such as identification of tentative molecular diagnosis from clinical data or genetic variant interpretation), major challenges persist in integrating multimodal data (genomic sequences, imaging, and clinical records) into unified and clinically robust pipelines, facing limitations in generalizability and practical implementation in clinical settings. This review provides a comprehensive classification and assessment of the current capabilities and limitations of LLMs in transforming hereditary disease diagnostics and supporting genetic education, serving as a guide to navigate this rapidly evolving field, while outlining application use cases, implementation guidance, and forward-looking research directions.

## 1 Introduction

### 1.1 Machine learning, deep learning, and language models

Machine learning (ML) has become a crucial tool in various fields, from healthcare to research, due to its ability to automate complex tasks and discover patterns in large datasets. Recent reviews highlight the growing impact of ML approaches in biomedical fields, including applications in diagnosing rare diseases and improving clinical outcomes (Manjurul Ahsan et al., 2022; Roman-Naranjo et al., 2023).

Traditional machine learning methods, such as decision trees and support vector machines, have been effective in solving well-defined problems where labeled data is abundant. However, these methods often struggle with high-dimensional data, complex relationships, and tasks that require context-dependent understanding, such as natural language processing (NLP) and genomics. One of the major challenges in traditional ML is handling large datasets with long-range dependencies–where information far apart in the data sequence needs to be considered together to make accurate predictions. Additionally, it often relies on manual feature extraction and struggles with tasks that require a deeper context or understanding of relationships across the data.

With the advent of deep learning (DL), many of these limitations were overcome. Deep learning, particularly with the use of neural networks, enables models to learn directly from raw data by automatically discovering useful patterns and representations. Convolutional Neural Networks (CNNs) excel at processing images (Alzubaidi et al., 2021), while Recurrent Neural Networks (RNNs) were initially used for sequential data like text (Mahmood Al-Selwi et al., 2024). However, RNNs also encountered difficulties with tasks that involved understanding relationships across long sequences of text due to their inherent sequential processing. This led to the development of transformer-based architectures, which revolutionized NLP and a range of other fields.

The introduction of transformer models in 2017 marked a significant breakthrough in deep learning (Vaswani et al., 2023). Unlike RNNs, transformers use an attention mechanism that allows the model to focus on different parts of the input data simultaneously, capturing long-range dependencies more effectively. This approach solves the problem of sequential processing and enables the model to understand complex relationships in data, very critical in healthcare and genomics. Transformers are particularly powerful in tasks that require context comprehension, such as text generation, translation, and named entity recognition. Their architecture consists of two main components: the encoder, which processes the input data (e.g., text or any other sequence, such as DNA), and the decoder, which generates the output (e.g., text). These terms refer to different stages of the model’s operation: encoding involves breaking down and analyzing input data to form a representation, while decoding reconstructs or predicts the next part of the sequence based on that representation.

BERT (Bidirectional Encoder Representations from Transformers) and GPT (Generative Pre-trained Transformer) are two of the most widely known transformer-based models, each tailored for different purposes. BERT is an encoder-only model, designed to understand text in both directions (left to right and right to left), which enables it to capture a more complete context for tasks like text classification and entity recognition. This bidirectional understanding allows the model to make more accurate predictions about the meaning of a word or phrase based on its surrounding context (Jacob et al., 2019). BERT outputs embeddings for the input, learned numeric vectors that represent a token, span, or the whole sequence; in BERT these embeddings are contextual: the vector for a word depends on its surrounding text, therefore semantically related items lie close in the embedding space and can be compared or fed to downstream classifiers. On the other hand, GPT is a decoder-only model that focuses on generating text, predicting each next word based on the preceding words in a unidirectional fashion. This makes GPT highly effective at tasks, such as text generation, translation, and summarization (Radford et al., 2018).

The ability of transformers to handle large datasets and maintain coherence over long sequences has led to the development of large language models (LLMs) - models with millions or billions of parameters (Brown et al., 2020). These models are capable of performing a variety of tasks by leveraging either full training on large datasets or fine-tuning with smaller, task-specific datasets. Fine-tuning allows the model to adapt to new tasks with minimal additional data, making few-shot or one-shot learning techniques possible: in few-shot learning, the model requires only a few labeled examples to perform well, while in one-shot learning, it can generalize from just a single example. This adaptability enables LLMs to be highly efficient across a range of applications, including research, healthcare, and education, without the need for retraining from scratch (Du et al., 2024; Zampatti et al., 2024; Aronson et al., 2024; Ueda et al., 2024; Laye and Wells, 2024).

Vision Transformers (ViTs) have further extended this approach beyond text, applying the transformer architecture to image processing tasks (Dosovitskiy et al., 2021). By treating image patches like words in a sentence, ViTs can capture dependencies across different parts of an image, making them highly effective in tasks like image classification and segmentation. The versatility of transformers across multiple domains demonstrates their power and adaptability, making them integral to modern Artificial Intelligence (AI) applications.

Generative Adversarial Networks (GANs) (Goodfellow et al., 2014) complement this landscape as specialized models for data generation, enabling the synthesis of highly realistic images, biomedical data, and even artificial genetic sequences through adversarial training.

Meanwhile, foundation models are trained on vast and diverse datasets and subsequently adapted (fine-tuned) to a wide variety of downstream tasks with minimal task-specific data. They shape the backbone of modern AI, providing general-purpose representations that can be adapted to a variety of specialized tasks. These models excel in transferring learned knowledge to new domains, accelerating advances in research, healthcare, and genomics.

Several approaches are commonly used alongside LLMs. The first is retrieval-augmented generation (RAG): before asking the model to answer, we first retrieve relevant passages from a curated corpus/database and pass them in as context. This grounding helps the model stay factual and cite evidence, because it reasons over the provided text rather than trained data (Lewis et al., 2021). The second approach involves the use of agents: instead of responding immediately, the model plans the steps, calls on tools (e.g., search engines, databases, calculators, code), inspects the results, and only then produces a response. This enables multi-step, up-to-date answers, but it works best with guardrails (whitelisted tools, sandboxing, logging) (Yao et al., 2023; Schick et al., 2023). We define these briefly here and analyze design patterns and failure modes in the Discussion.

### 1.2 Overview of human medical genomics

Medical genomics focuses on the application of genome analysis methods for the prevention, diagnosis, and personalized management of human diseases. The methodology, however, may vary depending on the type of disease in question. Thus, for Mendelian disorders, there are two principal tasks that are inherently interconnected: i. establishing the correct diagnosis of the disease or syndrome affecting the patient; and ii. finding the exact genetic cause(s) of the condition (reviewed in (Barbitoff et al., 2024)). The same two tasks are of paramount importance in cancer, where establishing the mutational profile of the tumor is essential for planning its treatment and prognosis. Another important area is the evaluation of the individual risk of the disease or specific clinical outcomes. Such prediction may be based on both genetic and environmental factors, and is especially relevant in cancer genomics and genomics of complex disease (Wand et al., 2021). Importantly, genome analysis is frequently not limited to genome sequencing or array-based genotyping, and may involve a rich set of functional genomics tools (e.g., gene expression analysis or epigenomic profiling), particularly in cancer genomics.

Regardless of the type of disease and methods used, the clinical genomic workflow can be partitioned into three stages, hereafter called pre-analytical, analytical, and post-analytical. This structure is aligned with the ISO 15189:2022 (Medical Laboratories, 2016), which formalizes the same sequence as pre-examination, examination, and post-examination processes. These stages or “phases of laboratory testing” encompass, respectively, test selection and specimen handling; test execution and interpretation; and report preparation, authorization, and delivery to clinicians (Fleming et al., 2017).

In the context of medical genomics, the pre-analytical stage comprises biospecimen collection, organization and preprocessing of clinical data, determination of tentative diagnosis, and selection of methods that will be used for genetic testing. The next (analytical) stage is the core diagnostic phase, where genomic data are generated, processed, and interpreted. Depending on the data type, their processing and interpretation may involve identification of causal genetic variants, gene expression changes, or other types of molecular biomarkers. As shall be noted later in this review, the collection of genomic data is sometimes omitted, and inference regarding genetic alterations is made on the basis of clinical data or other types of laboratory tests. Finally, the postanalytical phase focuses on communication of the genetic test results to the patient, further patient management and counseling.

While recent reviews have explored the potential of artificial intelligence and, more specifically, transformer models in healthcare and genomics, many have limitations in scope or model specificity. For example, some reviews focus solely on the applications of ChatGPT without a systematic analysis (Zampatti et al., 2024; Wang Jinge et al., 2024; Jeyaraman et al., 2023), making them outdated or too narrowly focused. Broader reviews, such as those on LLMs in general healthcare applications or in bioinformatics, lack a specific emphasis on genetic diagnostics (Bedi et al., 2025; Cheng, 2024; Lin et al., 2025). In parallel, a recent systematic review and meta-analysis comparing generative AI with physicians provides aggregate diagnostic accuracy estimates but is not focused on genetics as well (Takita et al., 2025). Some reviews are limited to a specific disease, such as dementia (Berrios Moya, 2024), oncology (Webster et al., 2023; Mudrik et al., 2024), schizophrenia (Deneault et al., 2024) and often does not have a clear emphasis on transformer-based models (Venkatapathappa et al., 2024; Xiang et al., 2024), moving a the scope of insights away from LLMs for genetic data analysis.

The closest topical review broadly covers AI in clinical genetics: it focuses on conventional DL methods and lacks depth on LLMs and transformers (Duong and Solomon, 2025). It is also not systematic or comprehensive, limiting its value as a foundational reference. This systematic review focuses specifically on the application of transformer models and generative AI in the research and diagnosis of hereditary diseases in recent years. To provide a comprehensive perspective, we reviewed models from four key sources: PubMed, bioRxiv, medRxiv, and arXiv, thus including both peer-reviewed studies and the latest preprint models. Since many state-of-the-art models are initially released as open-source in preprint repositories, this approach ensured we did not overlook recent developments. The growing need for efficient data processing and analysis in these domains highlights the potential of LLMs to revolutionize our understanding of genetic data, improve diagnoses, and predict disease outcomes. By exploring the use of LLMs in pre-analytical, analytical, and post-analytical stages, this review aims to provide systematic insights into how these models are transforming diagnostics, automating clinical processes, and supporting personalized medicine. A dedicated section will also assess the performance of models in clinical and research settings, examining both effective and problematic practices and ways to handle them.

Given the rapid release cycle of foundation and clinical LLMs, our goal is not to enumerate every method. Instead, we distill robust task patterns and workflows (e.g., extraction, retrieval-augmented generation, agentic pipelines, ViT-based and multimodal fusion), provide implementation guidance, and highlight near-term opportunities and risks for clinical deployment. Our search window covers publications up to 31 January 2025; later works are discussed selectively where they materially affect the argument.

## 2 Methods

**FIGURE 1:**
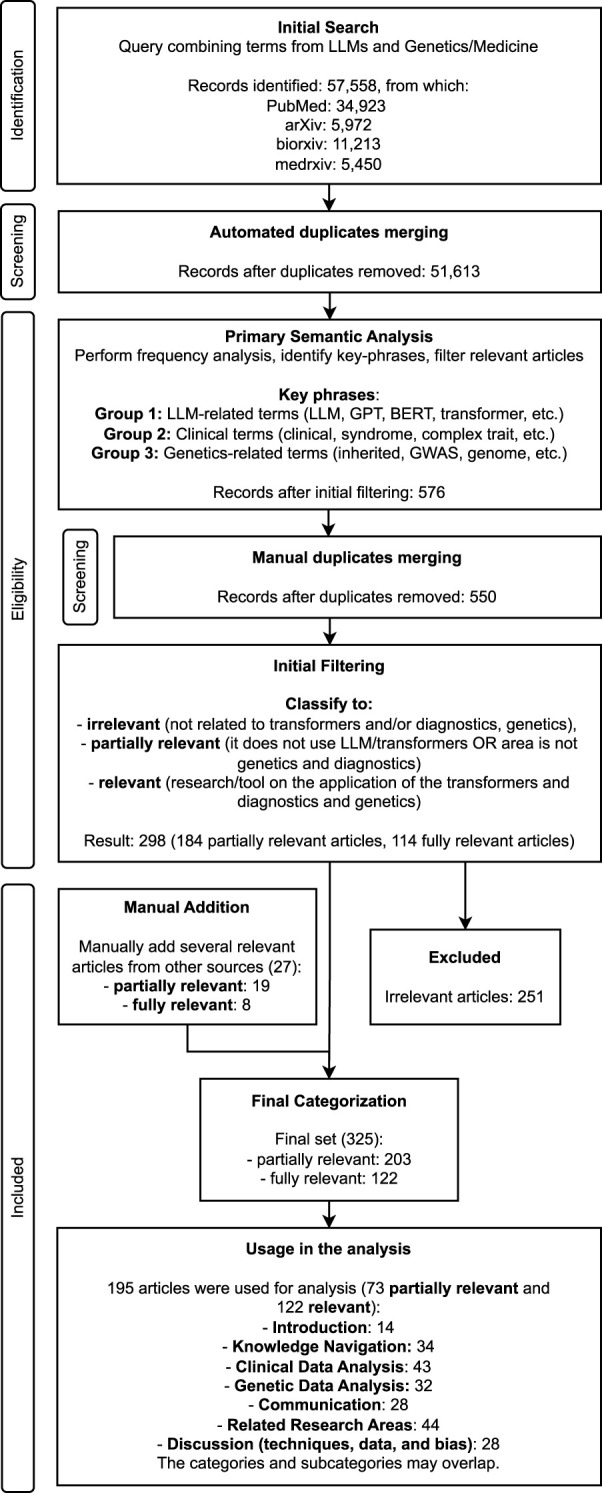
Pipeline of search strategy and filtering of the articles.

To comprehensively analyze the usage patterns of transformer-based models in genetics and hereditary diseases, a systematic review approach was developed according to the latest PRISMA 2020 guidelines for reporting systematic reviews (Page et al., 2021), ensuring thorough and transparent coverage of relevant studies. The search strategy was carefully constructed with selected terms relevant to transformer-based models and genetics, and all records were evaluated through a consultative process by two researchers, allowing for in-depth discussions on ambiguous cases, promoting a balanced selection, and reducing potential bias. The full search process is visualized in Figure 1.

### 2.1 Search strategy

To systematically review the use of LLMs in genetics and hereditary diseases, an initial broad search for relevant articles in English was conducted across multiple major scientific databases. A custom Python script was developed to automate the collection of articles from PubMed, bioRxiv, medRxiv, and arXiv (see *Data Availability* for access to the code repository). The search criteria focused on articles from 2023, 2024, and the beginning of 2025 (January) to ensure the inclusion of the most up-to-date research in this rapidly evolving field (the dataset was downloaded on 31-01-2025). Articles from medRxiv and bioRxiv were accessed through the API available at https://api.biorxiv.org/ (accessed on 31-01-2025), while arXiv data was retrieved using the Python wrapper https://github.com/lukasschwab/arxiv.py for the arXiv API (accessed on 31-01-2025). PubMed articles were accessed via the Biopython package for the PubMed API (C et al., 2009), available at https://biopython.org/docs/1.76/api/Bio.Entrez.html (accessed on 31-01-2025). This process yielded an initial dataset of 57,558 articles, forming the basis for further analysis.

The query terms were divided into two groups: one related to genetics and medicine, and the other related to transformer models and LLMs. Relevant articles were required to contain at least one term from each list in their title and/or abstract:

- genomic, genetic, inherited, hereditary, heredity, inheritance, heritability, disease subtype, NGS, next-generation sequencing, next-generation sequencing, genome sequencing, phenotype description, variant interpretation, complex trait, medicine, medical, diagnosis, diagnostic, clinical, clinical decision, syndrome.
- LLM, large language model, NLP, natural language processing, GPT, chatGPT, transformer, BERT, Bidirectional Encoder Representation, RAG, retrieval-augmented generation, retrieval augmented generation, generative AI, AI assistant, prompt, chatbot, prompt engineering, attention mechanism, chain-of-thought, chain of thought.

### 2.2 Inclusion and exclusion criteria

**FIGURE 2:**
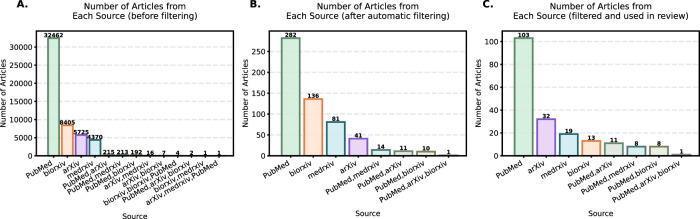
Distribution of articles by source: **(A)** after automatic deduplication and merging (51,613 records in total); **(B)** after additional automated filtering for relevance to clinical diagnostics (576 records in total); **(C)** final set used in this review (articles were manually curated, some of them were merged) (195 records in total).

After retrieving articles, several steps of filtering and exclusion were conducted. The first step in data processing involved automatically removing duplicate entries and cleaning the data, reducing the dataset to 51,613 articles. This was done using text processing algorithms to detect similarities in titles and abstracts. Figure 2A illustrates the contribution of each database to the final dataset, with a substantial number of preprints included. Although preprints offer access to the latest research, they lack peer review and may contain unverified results, requiring careful analysis.

A primary semantic analysis was performed to assess the relevance of each article to the research objectives. To identify domain-specific terminology during screening and curation, TF-IDF (Term Frequency-Inverse Document Frequency) scores were calculated for all words and phrases found in article titles and abstracts. This analysis was conducted at multiple levels: for the full corpus, for the selected set of articles, with generic AI/ML phrase filtering, and using a context-preserving fine-tuned approach (full methods, detailed results, and visualizations are in Appendix B and Supplementary Figures 1–4). This helped highlight key terms related to genetics, hereditary diseases, and LLMs. The identified phrases were grouped into three semantic categories:

- LLM-related terms: LLM, large language model, NLP, natural language processing, GPT, chatGPT, transformer, BERT, Bidirectional Encoder Representation, RAG, augmented generation, generative AI, AI assistant, prompt engineering, chatbot, prompt engineering, attention mechanism, chain-of-thought, chain of thought.
- Clinical terms: electronic health record, ehr, clinical, case report, cds, intensive care unit, medical, syndrome, phenotype, complex trait.
- Genetics-related terms: inherit, heredit, heritability, gwas, genome-wide, genome wide, association stud, snp, single nucleotide, genetic, variant interpretation, genomic varia, human gen, NGS, generation sequencing.

These key phrases were used to filter the articles based on the presence of at least one term from each group. To ensure coverage of morphologically derived forms (e.g., “inherited”, “genomics”, “associations”), the terms above were defined using stemmed substrings and matched via regular expressions. Filtering required that each article contain at least one match from each of the three categories.

To avoid false positives caused by accidental substring matches in unrelated words (e.g., “coverag” or “encourag” falsely matching “rag”), an empirically derived exclusion list was applied. This list was constructed by manually reviewing articles irrelevant to the study focus and identifying recurring misleading terms. This list included the following terms or common letter combinations: *tragic, fragment, coverag, encourag, ungs, angs, ongs, ings, eragrostis, smallmouth, fragile, angptl, intragenic, fragment, hallmark, uvrag, leverag, storag, averag, coverag, encourag, forage, liraglutid*. This filtering strategy significantly improved the precision of semantic classification by excluding structurally similar but contextually irrelevant terms. After the initial automated filtering step, the dataset contained 576 articles (Figure 2B).

Additionally, a manual verification step was conducted to identify and remove duplicate entries that were not detected automatically. In several cases, articles had slightly different titles or abstracts but were authored by the same group and described the same study. Based on this content-level similarity and author overlap, duplicates were removed, reducing the dataset from 576 to 550 articles for subsequent analysis. The complete list of included articles is provided in Supplementary Table 1. As previously noted, this step, as well as all subsequent ones, were conducted jointly by two researchers, allowing for careful discussion of ambiguous cases and minimizing potential bias.

After deduplication, articles were manually divided into three classes, based on their relevance to the topic. In order to be considered fully relevant (114 articles), the articles had to meet the following criteria: i. involve development or evaluation of transformer-based or similar models; and ii. focus on the extraction, processing, or prediction of genetic information or phenotypic information directly linked to inherited disease (e.g., recognition of rare disease symptoms). 184 articles met only one of these criteria (i.e., described non-transformer models or dealt with adjacent fields of research not directly linked to clinical genetic testing) and therefore were classified as partially relevant. All other articles (252) were considered irrelevant and were excluded.

In addition to automated filtering, 27 manually selected articles of partial (19) and high (8) relevance were included in the final dataset, bringing the total to 325 articles (Supplementary Table 2): 203 partially relevant articles and 122 fully relevant articles. Additional articles were sourced through references from the initially selected studies, as well as through further targeted filtering and searches across the originally extracted dataset.

**FIGURE 3:**
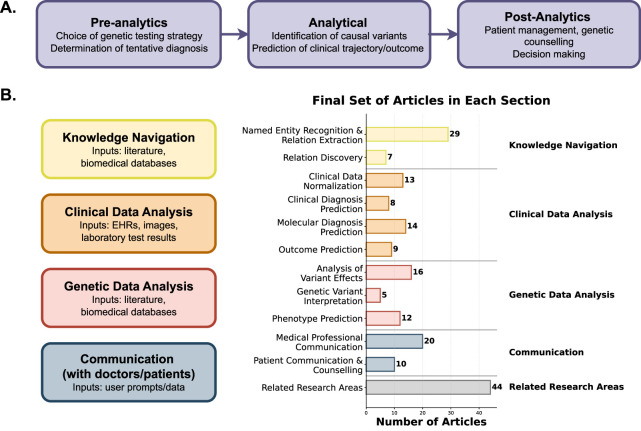
A diagram showing the applications of LLMs in the research and diagnosis of human genetic diseases. A diagram showing the applications of LLMs across stages of the genetic diagnostics workflow. **(A)** Pre-analytical, analytical, and post-analytical phases of clinical genetics, illustrating how LLMs support test selection, variant identification, and decision-making. **(B)** To the left: four major functional domains of LLM use: knowledge navigation, clinical data analysis, genetic data analysis, and communication with clinicians or patients. To the right: distribution of the final set of reviewed articles with corresponding subcategories highlighted.

At this stage, a thorough investigation of the selected articles was conducted. All highly relevant articles, as well as some of the partially relevant ones, were included in the analysis. From the latter category, only those entries were chosen that provided good examples of deep learning methods used in diagnostics, even if not specifically focused on LLMs or transformers. During the review process, each article was assessed based on its relevance to specific sections (see *Results*). Additionally, insights into best and worst practices of transformer usage are outlined in the discussion section. Since these areas encompass a broad list of tasks, they have been divided into specific applications, and itemized (see the relevant sections and Figure 3).

In total, of the 325 categorized studies, 195 were used for the analysis (Figure 2C): 122 relevant and 73 partially relevant. Among these, 154 focused on diagnostics and 27 were used as examples of practices discussed in the review (with some articles used in multiple sections). Furthermore, 14 articles were incorporated in the introduction as examples of existing systematic research with a similar topic (see Figures 1, 3B).

### 2.3 Risk of bias

A large proportion of the selected articles came from preprint databases, such as arXiv, bioRxiv, and medRxiv, meaning they had not yet undergone peer review. This could introduce some bias, as these studies have not been validated by the scientific community. However, given the fast-paced nature of LLM development, many of the most cutting-edge techniques are being developed faster than the peer-review process allows. Consequently, it was deemed essential to include such articles to capture the most current advancements.

Additionally, while this review focuses on the application of LLMs in the specific domain of genetics and hereditary diseases, there may be general-purpose models or methods from broader AI fields that were not included in this focused analysis. These models could still provide valuable insights or advancements applicable to this domain, although they fall outside the scope of this particular review.

### 2.4 Semantic landscape of the literature (TF-IDF)

To characterize the semantic landscape of the literature, we used TF-IDF (term frequency-inverse document frequency), which emphasizes terms that are frequent within documents but relatively distinctive across the corpus. TF-IDF profiling showed a consistent progression from generic to domain-specific themes (Supplementary Figure 1). In the full corpus ( 51,613 articles; Supplementary Figure 1A), generic phrases such as “language models”, “large language”, and “artificial intelligence” dominated, confirming broad field coverage prior to curation. The curated set (195 articles; Supplementary Figure 1B) preserved these anchors and surfaced domain cues (e.g., “precision medicine”): evidence that selection retained the core landscape. After removing generic AI/ML phrases (Supplementary Figures 1C, 3), specific trends emerged, including “precision medicine”, “gene expression”, “open source”, and “genetic testing”, alongside disease-focused (“breast cancer”, “alzheimer disease”), resource-oriented (“human phenotype ontology”), and technique-oriented (“attention mechanism”, “single cell”) terms.

A context-preserving fine-tuned analysis was performed to address potential concerns about removing AI/ML terminology (Supplementary Figure 4). This approach first trained the TF-IDF model on the curated dataset with full vocabulary, then applied *post hoc* reweighting to down-weight generic terms while preserving semantic relationships. The fine-tuned analysis confirmed that domain-specific trends remain stable across different filtering strategies, validating our findings.

Source comparisons (Supplementary Figures 2–4) further clarified complementarity. Before filtering, PubMed ( n=131 ) and preprints ( n=64 ) shared 57% of top phrases ( 17/30 ), indicating strong consensus on core topics. After filtering, overlap dropped to 23% ( 7/30 ), revealing distinct emphases (Supplementary Figure 3). The fine-tuned analysis showed similar patterns with 23% overlap ( 7/30 ), confirming the robustness of these findings.

PubMed leaned clinical and translational (“precision medicine”, “genetic testing”; established disease terms), whereas preprints highlighted emerging computational motifs (“gene expression”, “open source”, “attention mechanism”) and method-forward phrasing. Many key terms from both technical and biological domains ranked highly in preprints but were absent from PubMed, supporting our dual-source strategy and underscoring that inclusion of preprints offers a more comprehensive view of the field.

Topic modeling further revealed the semantic structure of the literature (Supplementary Figure 5). Eight latent topics were extracted using Latent Dirichlet Allocation (LDA), capturing distinct research themes from clinical variant interpretation to computational method development. The topic overlap visualization demonstrates how different research areas interconnect, with some topics (e.g., clinical diagnostics and precision medicine) closely related while others (e.g., protein structure prediction and variant calling) occupy distinct semantic spaces.

## 3 Results

Our systematic review identified and used a total of 195 studies that report application of generative AI methods for a wide variety of tasks within the scope of human medical genomics. After careful curation, we have split these studies into four main categories depending on the study design, methods and data types employed: i. knowledge navigation (34 articles); ii. clinical data analysis (43 articles); iii. genetic data analysis (32 articles); and iv. communication with patients and medical professionals (28 articles). Each category was then subdivided into several subcategories corresponding to major tasks addressed by respective generative AI methods and models (Figure 3B). In the following sections, we will summarize articles from each category, highlighting the most notable studies and discussing prospects for further method development in each area.

### 3.1 Knowledge navigation

Most of the studies in the knowledge navigation category dealt with the extraction of structured information from published sources or biomedical databases. In many cases, this goal is achieved through named entity recognition (NER) and relation extraction (RE), and is primarily focused on the extraction of gene-disease or variant-disease relationships from published literature (e.g., (Huang Dao-Ling et al., 2024)). This task is exceptionally important given the vast amount of such information available in the literature, which is, in many cases, not properly reflected in major databases such as Online Mendelian Inheritance in Man (OMIM) (Hamosh et al., 2005) or NCBI ClinVar (Landrum et al., 2017). Data extracted from literature sources are crucial for clinical geneticists and can be used at all stages of the genetic testing workflow. Thus, knowledge about gene-disease associations may aid in the selection of appropriate genetic testing methods and inform interpretation of sequencing results. Complementing these trends, training very small, task-specific encoders is emerging as an accuracy-preserving and controllable alternative to general LLMs, highlighting the promise of small, fine-tunable models for biomedical tasks while reducing hallucination risk (Saha et al., 2025). At the same time, there is a rising trend of employing decoder-based LLMs (e.g., GPT-3.5/4, PhenoGPT, GP-GPT) for entity-level tasks, which, despite convenient one/few-shot use (providing one to several examples right in the query) and promising results in some studies, may be suboptimal for extraction tasks due to architectural mismatches (Murphy et al., 2024; Lyu et al., 2024). This trend invites further research, as will be described in the Discussion.

Beyond the extraction of simple relations, some studies involved a more sophisticated design. For example, some studies focused on complex multi-entity relationships (e.g., DUVEL (Detection of Unique Variant Ensembles in Literature) (Nachtegael et al., 2024)). In other studies, the extraction of gene-disease associations was complemented with curated resources and interpretable extraction frameworks (e.g., GPAD (Gene-Phenotype Association Discovery), RelCurator (Tahsin Hassan Rahit et al., 2024; Lee et al., 2023)). Several works combined knowledge navigation tasks with question answering, developing specialized tools and models for interactive communication with researchers or clinicians. Examples of such Q&A systems include PubTator 3.0 (Wei et al., 2024) and BioMedLM (Bolton et al., 2024), and demonstrate improved answer factuality and superior performance compared to general-purpose LLMs. A number of specialized systems, such as ClinVar-BERT, AutoPM3, and VarChat, are optimized specifically for variant interpretation, providing variant impact summaries (De Paoli et al., 2024) or extracting pathogenicity evidence for genetic variants from published sources (Li Weijiang et al., 2024; Li Shumin et al., 2024).

Aside from the 29 studies involving information extraction, a separate subcategory (comprising 7 studies) focused on the prediction of novel gene-disease relationships. These studies utilized both models specifically trained for solving the task of causal link prediction (e.g., end-to-end disease-gene association prediction model with parallel graph transformer network (DGP-PGTN) (Li Ya et al., 2023) or LitGene (Jararweh et al., 2024)) as well as interactive large language models (e.g., Med-PaLM 2 (Tu et al., 2023)). Another notable work described the application of transformer-based models to the identification of causal genes at GWAS loci (Shringarpure et al., 2024). While limited in number, these studies illustrate the potential of generative AI methods for hypothesis generation–a goal which, if successfully met, can greatly advance biomedical research in various fields beyond medical genetics.

### 3.2 Clinical data analysis

This category was the largest in our analysis and comprised diverse efforts involving the analysis of electronic health records (EHRs), clinical notes, and results of non-genetic laboratory testing with a goal of phenotypic data organization, providing tentative diagnosis or disease subtypes. Similarly to literature review, these types of analysis are more commonly performed prior to or during genetic testing with a goal of selecting the appropriate testing strategy and enhancing interpretation. However, as shall be described below, there are several attempts to circumvent the need for genetic testing by providing information on actionable genetic markers based solely on other types of data.

The first subcategory of studies focused on extraction and normalization of clinical information from EHRs. Methods employed for this task largely overlap with those used for extraction of information from scientific literature. In purview of clinical data processing, however, the main emphasis is laid onto the extraction and normalization of phenotypic information of the patient, typically by mapping it onto Human Phenotype Ontology (HPO) terms (Gargano et al., 2024) using both encoder- and decoder-based models (Yang Jingye et al., 2024; Albayrak et al., 2025; Murphy et al., 2024; Soysal and Roberts, 2024; Hier et al., 2025; Weissenbacher et al., 2024).

Beyond normalization of phenotype descriptors, a large number of models are built for suggesting genetic diagnosis on the basis of the patient’s phenotypic features using textual (EHRs) or visual information (e.g., portrait photos or data from other imaging methods). In the former category, generative LLMs such as GPT-3.5, GPT-4, and Gemini (which are obsolete at the moment) have been applied to suggest candidate diagnoses in autoinflammatory and neurogenetic disorders, or predict cancer predisposition genes from textual EHR summaries (Pillai and Pillai, 2023; Zampatti et al., 2025; Sultan et al., 2023). Models with visual data inputs are also designed to predict both tentative diagnoses and causal genetic alterations. For example, an older CNN-based model called DeepGestalt has proven its efficacy in syndromic features identification (Gurovich et al., 2019), with its newer version, GestaltMML (multimodal Transformers over facial photos, clinical notes, and metadata), having improved accuracy due to its multimodal design (Wu et al., 2024). In oncology, a large number of models have been built to predict the mutational profile of the tumor based on histopathology data (whole slide images, WSIs). Examples of such efforts include prediction of gene mutation status (Guo et al., 2023; Huang Gexin et al., 2024; Sun et al., 2024; Singh et al., 2024; Akram et al., 2024; Guo et al., 2024) or aggregate genomic features such as tumor mutational burden (Wang et al., 2025). A peculiar feature of these approaches is that they are designed as a substitute for, rather than being complementary to, costly genetic testing.

Finally, a series of studies focused on the prediction of various clinical outcomes in patients using a mixture of genetic and non-genetic information. Notable examples of such studies include stratification of survival risk in breast cancer patients (Kumar Mondol et al., 2024) or genetics-informed subtyping of Alzheimer’s disease patients (Machado Reyes et al., 2024a).

### 3.3 Genetic data analysis

While bioinformatic analysis of genomic data is commonly considered to be the most complicated step of a medical genomics workflow, only a minority of studies identified by our review directly employed generative AI for genetic variation analysis. The respective methods were focused on three major tasks: i. phenotype-agnostic prediction of functional impact of genetic variants; ii. prioritization of genetic variants in the context of NGS results interpretation; and iii. aggregation of genetic variation data for prediction of the patient’s phenotype (typically, in connection with complex disease).

In the first subcategory, much of the promise of generative AI is connected with the development of domain-specific models (foundation models) to understand the language of biological molecules (e.g., DNA or proteins). While biological sequences differ from natural language due to a lack of easily identifiable “words”, AI methods have already demonstrated their extraordinary capabilities in solving fundamental tasks. Nobel prize-winning AlphaFold (Jumper et al., 2021) is the most notable example of such models that demonstrated groundbreaking performance in protein folding. A number of well-established methods have been developed on top of the protein language model employed by AlphaFold, including AlphaMissense, a tool that has become a *de facto* gold standard in the evaluation of pathogenicity of amino acid substitutions (Cheng et al., 2023). Beyond prediction of impact for amino acid substitutions in proteins, a range of models for working with DNA sequence have been proposed, with some showing promising results in tasks related to genetic variation analysis (e.g., prediction of splice sites, epigenetic marks, enhancer sequence, promoter sequence, enhancer activity, chromatin profile, and others) (Ying et al., 2024; Dalla-Torre et al., 2025; Li Hongyang et al., 2025; Machado Reyes et al., 2024b). These models are already trained to understand the context of a sequence, and their representations can be fine-tuned for a diverse range of downstream tasks. In addition, models trained for specific tasks also exist–their advantage is that they can be much smaller and therefore require fewer computational resources. In a notable study, transformers have advanced splice site prediction for identifying disease-relevant splice variants (Jónsson et al., 2024). Transformer-based variant annotation is not limited to point mutations - for instance, a tool called PhenoSV applies attention-based modeling to structural variants (SVs) to capture how both non-coding and coding structural variants affect gene function (Xu Zhuoran et al., 2023).

Another important and particularly challenging area of bioinformatic analysis of genome sequencing data is the identification of causal genetic variants among millions present in each individual genome (Barbitoff et al., 2024). In this field, a variety of generative AI methods have also shown their exceptional performance. For example, authors of the Mendelian Approach to Variant Effect pRedICtion (MAVERICK) tool report ranking the causal variant among the top five variants in over 95% of the cases (Danzi et al., 2023). Other tools, such as Genetic Transformer (GeneT), also report high performance in variant prioritization (Liang et al., 2024), and benchmarking studies confirm substantial improvement of clinical variant classification from using state-of-the-art models and other techniques, such as fine-tuning and RAG (for details see discussion), including LLMs (Boulaimen et al., 2024; Lu and Cosgun, 2025).

Finally, a set of generative AI-based methods has been developed to enhance polygenic risk prediction in complex diseases. A recurrent strategy employed in several studies is the application of LLMs and other models for the construction of informative predictive features (such as epigenetic markers) based on the individual genotypes (e.g., Epi-PRS (Zeng et al., 2024) or epiBrainLLM (Liu et al., 2024)). Other studies attempt to use transformer architectures for modeling epistatic interactions between genes (Lee et al., 2024) or for simple classification of patients into subtypes based on their genotype, as exemplified by a study in Parkinson’s disease (Machado Reyes et al., 2022).

### 3.4 Interaction with patients and medical professionals

The last category of generative AI applications in medical genetics leverages the unique capacity of LLMs to communicate with the user in natural language. Such communication typically involves medical question answering, and can assist both medical professionals and patients. As mentioned in previous sections, interactive chatbots have been developed and used for various tasks mentioned earlier in this review, including knowledge navigation, clinical data analysis, and genetic variant interpretation (Ali Quidwai and Lagana, 2024; Nat et al., 2024; De Paoli et al., 2024; Sultan et al., 2023; Zampatti et al., 2025; Lukac et al., 2023; Hewitt et al., 2024; Hamilton et al., 2024). However, the use of generative AI for interaction with researchers, doctors, and patients is not limited to Q&A tasks. In this subsection, we will briefly describe other notable works involving communication with patients or medical professionals.

In the realm of interaction with medical professionals, one study has reported the use of generative AI to address privacy challenges of using real patient images in genetics education. A study on Kabuki and Noonan syndromes found that AI-generated facial images, created using StyleGAN (Karras et al., 2019) methods, were nearly as effective as real photos in training pediatric residents to recognize phenotypic features (Waikel et al., 2024). While real images were rated slightly more helpful, synthetic ones notably increased diagnostic confidence and reduced uncertainty.

Some works are focused on the development of interactive assistants for the interpretation of genetic test results. One notable example is the study by Yang Jiaxin et al. (2024) who have constructed an LLM module for textual summaries of submodules of a knowledge graph. Another notable case is the Just-DNA-seq platform that integrates a custom GPT model called GeneticsGenie to facilitate the interpretation of genetic test results by users with no genetics background (Anton et al., 2024). In another effort, an AI assistant was developed for the interpretation of pharmacogenomic test results (Murugan et al., 2024). Besides interpretation of genetic testing results, a range of studies have explored the application of LLMs in genetics question answering (Ke et al., 2025; McGrath et al., 2024), counseling (Fukushima et al., 2025; Patel et al., 2024), and education (Waikel et al., 2024; Walton et al., 2023). It has to be noted, however, that studies reveal variability in accuracy, especially in nuanced topics, such as inheritance patterns or ethical subtleties of genetic risk communication (McGrath et al., 2024; Walton et al., 2023). Besides, models still risk hallucination and outdated references, highlighting the need for oversight and continual retraining (Walton et al., 2023).

Taken together, all of the aforementioned applications are well aligned with general trends in the field of generative AI methods, which are increasingly being used as personal assistants in various fields. However, a range of technical and ethical concerns still raise doubts regarding the implementation of LLMs in clinical genetics in the near future (see Discussion for a more in-depth analysis of the outstanding issues).

### 3.5 Related research areas

Although this review focuses on the applications of generative AI models in human genetics and diagnostics, several adjacent research areas, while not directly related to human genome analysis, offer valuable insights and transferable lessons. These applications were excluded from the main focus due to limited direct relevance; however, they highlight the variety of possible usage across biological and medical domains and may inspire future applications in human genetic research.

Studies applying LLMs to microbial genomes have demonstrated the potential of language models to encode meaningful representations of whole genomes. For example, models trained on bacterial or fungal species can predict traits such as antibiotic resistance or habitat specificity (Naidenov and Chen, 2024; Li Zhufeng et al., 2025; Weinstock et al., 2024). While distinct from human genetics, these works show how transformer-based models can capture population structure and gene interactions in complex biological systems.

Transformer models have also been applied to protein sequences for predicting gene ontology terms and functional annotations (Tamir and Yuan, 2024). These studies operate in the space of proteomics, yet demonstrate modeling principles that could be extended to human gene function prediction or variant interpretation.

A substantial body of work with ViTs focuses on cancer imaging, particularly for tasks such as tumor segmentation, subtype classification, and spatial analysis from whole-slide images (Li Yin et al., 2023; Pizurica et al., 2024; Hu et al., 2024; Karim et al., 2024; Hillis et al., 2024; Yang Ping et al., 2024). While not always grounded in genomic data, these tasks intersect with genetic diagnostics when molecular subtypes play a role in treatment stratification.

Epigenetic regulation and cross-species prediction of gene expression using sequential and imaging data represent another promising direction (Weinstock et al., 2024; Ramprasad et al., 2024; Pizurica et al., 2024). These studies explore how attention-based models can generalize across evolutionary distances, enabling predictions in under-characterized organisms and informing functional annotation pipelines.

LLMs are being increasingly integrated into gene editing workflows: from automating guide RNA design and protocol generation (e.g., CRISPR-GPT) to predicting cellular responses to perturbations at single-cell resolution (e.g., scLAMBDA) (Huang Kaixuan et al., 2024; Wang Gefei et al., 2024). Together, these applications illustrate how transformer models support both the interpretation and manipulation of gene function in human genetics.

Transformer models have also been used to investigate the role of genetic support in the success or failure of clinical trials (Razuvayevskaya et al., 2024). While not directly diagnostic, such applications emphasize the growing role of human genetic evidence in pharmaceutical development and clinical decision-making.

Taken together, these diverse research directions extend the scope of transformer-based models beyond traditional genetics. By leveraging techniques and datasets from related fields, such as microbial biology, cancer diagnostics, and synthetic biology, future work in human genetics may benefit from models and insights developed in adjacent domains.

## 4 Discussion

As transformer models are increasingly integrated into specialized fields such as genetics, it is essential to evaluate their strengths and limitations. This research systematically reviewed the application of these models in genetic diagnostics, incorporating resources from PubMed, bioRxiv, medRxiv, and arXiv to ensure both peer-reviewed depth and inclusion of the latest model developments, focusing on hereditary disease diagnostics while retaining adjacent work for context.

In this section, we aim to provide a guideline for selecting the models and strategies for researchers willing to integrate generative AI tools in their workflows. We list and discuss the main benefits and limitations of different models and techniques, and highlight key developments needed to ensure the reliability and trustworthiness of LLM applications. We also touch upon emerging trends and techniques that may enhance their effectiveness.

### 4.1 Model selection guide

In this subsection, we summarize major families of generative AI models across a range of data types they process (modalities), including text, sequence, images, and their combinations (Table 1). We try to link the models to respective tasks and examples reviewed in the previous section.

**TABLE 1:** Architectures and capabilities map for genetics and adjacent tasks.

Family	Modality (examples)	Proper application	Limitations/cautions	Examples
Encoder-only	Text (clinical notes, biomedical literature)	NER/RE, mapping of terms to standardized vocabularies (ontologies)	Long documents may truncate, weak for free-form generation	ClinVar-BERT (Li et al., 2024a), PathoBERT (Rai et al., 2024), Big Bird (Zaheer et al., 2020)
Decoder-only	Text (clinical narratives, reports, instructions)	Drafting reports, Q&A, guideline summarization, next event prediction	Hallucinations without retrieval, version drift	GPT (Radford et al., 2018), ChatGPT (Brown et al., 2020), GeneGPT (Jin et al., 2024), Comet (Waxler et al., 2025)
Encoder-decoder	Text (summaries, structured templates)	Summarization, controlled generation (using templates)	All limitations of encoder- and decoder-only	T5 (Raffel et al., 2023)
Foundation models	Biological sequences (DNA, RNA, protein)	Tasks involving sequence analysis (e.g., variant and regulatory effect prediction, epigenomic signal transfer)	Miss long-range effects, less reliable for rare or cross-species data; require validation	GENA-LM (Fishman et al., 2023), Nucleotide Transformer (Ramprasad et al., 2024), MethylGPT (Ying et al., 2024), AlphaMissense (Cheng et al., 2023), MIPPI (Liu et al., 2023a), CellFM (Zeng et al., 2025), Enformer (Ramprasad et al., 2024)
ViT, Hybrid CNN-Transformer	Images (MRI, WSI, facial phenotypes)	Predictions based on imaging data (e.g., disease subtyping, prediction of genetic alterations)	Sensitive to data quality and bias, require expert annotation, limited by ethical constraints	CroMAM (Guo et al., 2024), BPGT (Huang et al., 2024b), PromptBio (Zhang et al., 2024a), ChromTR (Xia et al., 2024), Tokensome (Zhang et al., 2024b), GestaltMML (Wu et al., 2024), TSCA-Net (Fu et al., 2024)
Multimodal models	Multimodal (imaging, genomics, text, tabular data)	Integration of diverse data types	Modality imbalance; missing-modality handling	MGI (Zhou et al., 2024), BioFusionNet (Kumar Mondol et al., 2024), Genetic InfoMax (Xie et al., 2023)

Models based on transformer architecture (including encoder-only, decoder-only, and encoder–decoder variants) can be used to address a wide range of tasks involving biomedical text processing. Thus, encoder-style models (e.g., BioBERT, ClinVar-BERT, PathoBERT, Big Bird (Zaheer et al., 2020)) remain the most reliable for structured extraction and annotation (NER/RE, HPO normalization), and for structuring clinical data or texts. Decoder LLMs (including both general-purpose, such as ChatGPT, or specialized, such as GeneGPT) add value for generative tasks, such as clinician-facing Q&A, report drafting, and exploratory hypothesis generation, but typically require retrieval and tool calling for robust, auditable performance; mixed systems (RAG, agents–described in the next section) reduce hallucinations when grounded in curated resources and databases. Additionally, decoder-only models are applied not only for text generation, but also for predicting more complex entities, including next medical events (e.g., Comet (Waxler et al., 2025)). Lastly, full encoder-decoder (seq2seq) architectures are useful for controlled, template-constrained generation of text (e.g., highly structured summaries or other tasks). Some studies use decoder-only models (e.g., ChatGPT) where a transformer encoder would likely perform better for extraction-focused workloads (Shringarpure et al., 2024; Labbe et al., 2023; Hier et al., 2024). It is important to note that choosing the right architecture is critical. Although early comparisons often focused on GPT-3.5 vs. GPT-4, newer models and configurations combining reasoning and tool-use have been released. While they can improve results, studies consistently report hallucinations, outdated knowledge, and stylistic artifacts distinguishable from expert writing (McGrath et al., 2024; Patel et al., 2024; Hier et al., 2024; Hulman et al., 2023).

Foundation models for DNA/RNA/protein (e.g., GENA-LM, Nucleotide Transformer, MethylGPT, CellFM (Zeng et al., 2025), Enformer (Avsec et al., 2021)) provide reusable representations for splice/regulatory effect prediction, epigenomic signal transfer, variant effect scoring, and downstream tasks including drug response and trait/PRS modeling. Such pre-trained models can be fine-tuned for any specific task, provided with data. Their main cautions concern tokenization granularity, long-range dependencies, domain/species shift, and calibration on rare regions or reliance on predicted structures.

If the goal is to process other types of data beyond text or sequences, specialized model types have to be used. For instance, vision backbones and hybrid CNN-Transformer systems address a range of image processing tasks, including working with MRI, microscopic images, and facial phenotypes for tasks such as mutation status prediction (e.g., CroMAM, BPGT, PromptBio), karyotyping (e.g., ChromTR, Tokensome), and syndrome suggestion (e.g., GestaltMML). When the foal is to align or fuse imaging, sequences, or text, multimodal models (e.g., MGI, BioFusionNet, Genetic InfoMax) can be developed (see next subsection for discussion of related techniques). As mentioned in earlier sections, these models allow for more accurate predictions compared to single-modality models (e.g., for tasks such as predicting cancer patient survival (Kumar Mondol et al., 2024)). However, gains from using image data or multimodal fusion depend more on data acquisition methods (e.g., imaging instruments or staining techniques), and may be particularly vulnerable to class balance or other issues characteristic of traditional machine learning frameworks. These problems may have a comparable or even more dramatic impact than model size, and special attention has to be dedicated to data preparation. Techniques, such as normalization, site-balanced splits, and external validation mitigate common risks.

Finally, complex specialized architectures (Epi-PRS, Prophet, TransBTS) combine multiple mechanisms (convolution, attention, classical ML) to model relations across sequences, images, and text. Such pipelines can substantially improve machine understanding of clinical-genetic signals but require deeper domain knowledge in model training and testing.

Overall, model selection should be driven by task, data, and safety requirements: encoders for extraction and normalization, decoder LLMs (with retrieval/tools) for controllable generation, encoder-decoder models for structured seq2seq outputs, biological foundation models when specific pattern understanding is needed, and multimodal/vision architectures where phenotype-genotype links are image-mediated. Using the latest versions, domain adaptation, and careful prompting improves performance, but rigorous evaluation remains essential (Mondillo et al., 2024; Hewitt et al., 2024; Zampatti et al., 2025; Temsah et al., 2024). In the next section, we will consider techniques that can improve the results of model usage.

### 4.2 Model strategies

We now discuss how to achieve effective use of transformer models, since outcomes depend on how systems are composed. Table 2 aggregates prompting, retrieval, tool-use, long-context modeling, multimodal fusion, privacy-preserving training, and evaluation patterns, indicating when to use them, expected benefits, typical limitations, and concrete mitigations.

**TABLE 2:** From clinical data problems to LLM-based solutions: techniques/patterns with benefits, limitations, and mitigations.

Problem or task	Technique/ Pattern	Benefits	Limitations	Mitigations	Example models
Noisy or unstandardized data	Modality-specific preprocessing and QC	Cleaner input, higher signal-to-noise ratio	Sensitive to small changes, reproducibility risk	Standardized workflows and QC protocols, external validation	Chahid et al. (2023), Castro et al. (2020)
Local or data-specific patterns	Fine-tuning or domain adaptation	Higher accuracy on small, focused datasets	Overfitting, loss of general knowledge	Lightweight fine-tuning, frozen backbone, external cohort testing	LoRA (Hu et al., 2021)/adapters (Poth et al., 2023)/QLoRA (Dettmers et al., 2023)
Long-range dependencies	Long-context transformers	Distal genomic or textual relations captured	Tokenization-biology mismatch, distal trade-offs	Combine local and global contexts, add task-specific layers, benchmark against short-range models	GENA-LM (Fishman et al., 2023), Nucleotide Transformer (Dalla-Torre et al., 2025)
Multimodal or missing inputs	Contrastive learning or cross-attention fusion	Usage of complementary signals, greater robustness	Modality imbalance, missing input at inference	Hyperparameters tuning, validate on diverse, multi-site data	CroMAM (Guo et al., 2024), BioFusionNet (Kumar Mondol et al., 2024)
Need for structured reasoning	Prompting, Chain-of-Thoughts, one/few-shot	Consistent reasoning, reusable templates	Prompt leakage, verbosity, unstable zero-shot behavior	Few-shot verified prompts, separate reasoning/final output, regular review	BioGPT (Jin et al., 2024), Med-PaLM 2 Pati et al., 2024
Need for reference-grounded answers	Retrieval Augmented Generation (RAG)	Reduced hallucinations, improved factual grounding	Weak retrieval, outdated sources	Curated indices, freshness policies, inline citations	GeneGPT and others (Lu and Cosgun, 2025; Jin et al., 2024)
Need for tool or API execution	Agentic AI	Automated tasks decomposition and workflow execution	Tool errors, latency, unsafe calls	Restriction to verified tools, safety checks, human oversight	BioAgents (Mehandru et al., 2025), BioChatter (Lobentanzer et al., 2025)
Data privacy protection	Federated Learning	Collaboration without raw data sharing	Complex setup, coordination overhead	Site-specific evaluation plans, standardized protocols	SF-GWAS (Cho et al., 2025)
Fair testing and leakage prevention	Evaluation- or leakage-aware benchmarks	Transparent comparison, contamination control	Hidden leakage, overfitting to test data	External test sets, preregister benchmarks, no train/test overlap	CARDBiomedBench (Bianchi et al., 2025)

Prior to model training, data quality control (QC) and preprocessing are important. Artifacts, missing data points, or inconsistent phenotype capture degrade model inputs (Chahid et al., 2023; Castro et al., 2020; Nijman et al., 2022; Liu Mingxuan et al., 2023). Preprocessing (e.g., segmentation, standardization) improves data quality by reducing artifacts, but increases can also encode hidden bias or lead to reproducibility issues (Chahid et al., 2023; Castro et al., 2020). Fully documenting steps and QC, pinning versions, and testing on external datasets are essential for robustness and reproducibility.

General Language Models (e.g., BioBERT, GENA-LM, GPT-4) often miss disease- or site-specific patterns, labeled cohorts are small (risk of overfitting/forgetting), therefore models must be adapted for the target task while remaining flexible (Pati et al., 2024; Rockensc et al., 2025; Durkie et al., 2024). One of the possible solutions is using fine-tuning and domain adaptation. Full fine-tuning (i.e., task-specific re-training) can maximize alignment when labels and compute suffice, but risks overfitting and forgetting on small cohorts. Parameter-efficient methods (PEFT–small parameter updates (Xu Lingling et al., 2023); e.g., LoRA (Hu et al., 2021)/adapters (Poth et al., 2023)/QLoRA (Dettmers et al., 2023)) keep the backbone of the model frozen, reduce compute and exposure of protected information, and enable rapid iteration across disease/task variants (Dettmers et al., 2023). Mixed and continual training (keeping learning gradually) helps retain broad knowledge useful for retrieval and classification while still specializing: by exposing the model to diverse data/tasks at once, it builds more general representations, reduces overfitting, and stays flexible. In contrast, the classic pretrain-then-finetune pipeline deepens task-specific skills but is more prone to overfitting on small cohorts. Evidence suggests mixed training yields models that remain adaptable for downstream tasks, important in genetics, where knowledge and guidelines evolve, balancing specialization and generalization, e.g., for genetic counseling or variant interpretation (Allen-Zhu and Li, 2024). Recent findings also show that domain-specific pretraining alone does not guarantee superiority: randomly initialized models can match or exceed genomic foundation models in downstream tasks (Vishniakov et al., 2024). Moreover, very small, task-focused LMs with selective incremental learning can be competitive for pathway inference while reducing hallucinations (Saha et al., 2025).

Clinical notes and molecular sequences can be very long, thus contain distal dependencies that short-context models miss; moreover, the tokenization scheme (how sequences are discretized) and splitting into smaller chunks to fit a fixed context window can also fail to properly reflect biological functions. Long-context sequence models (e.g., GENA-LM, Nucleotide Transformer) deal with this by targeting both long- and short-distal dependencies in long notes and genomes. Hybrid windows (local+global), task-specific heads, and comparisons to short-context baselines help maintain accuracy.

What is more, decisions and solutions should often rely on data with multiple modalities, such as images, genomics, and text (otherwise, insights from complementary signals will be lost). There are two common ways for multimodal fusion, which means combining such data. The first approach is contrastive learning to place all modalities in the same shared space and train a model to understand the relationship between data points by learning to differentiate between similar and dissimilar pairs (Chen et al., 2020; Moghaddam et al., 2024). The second one is using cross-attention between modalities or late fusion to let one modality use information from another (Li and Tang, 2025). Typical problems are modality imbalance, a missing modality at test time, and domain shift from site/scanner/stain differences. Practical fixes include curriculum learning and hyperparameters (e.g., temperature) tuning (for contrastive), missing-modality heads, or modality dropout.

Additionally, specialists often need structured, step-wise outputs without retraining; naive prompts can leak context, get verbose, and behave unstably in a zero-shot setting (when output examples are not included in the query prompt). Prompting, including adding specific instructions for reasoning (Chain-of-Thought) (Lee et al., 2025; van Uhm et al., 2024), providing one to several examples of the desired structure and result (this technique is called one or few-shot learning), helps to create structured and desired outputs. However, it is still vulnerable to leakage and verbosity, therefore periodically validating/updating prompt libraries remains important.

Furthermore, clinical answers must be fact-checked and reference-backed, since relying on internal model memory can produce hallucinations (confident mistakes). RAG searches databases and websites before generating answers, therefore reducing hallucinations and providing a controllable, citable trace. Key practices include using curated indices (e.g., ClinVar/OMIM/HPO), enforcing freshness policies, applying document-grounded scoring, requiring inline citations, and using deterministic decoding with version pinning to ensure actuality, auditability, and stability (Coen et al., 2024; Murugan et al., 2024; Fukushima et al., 2025; Ali Quidwai and Lagana, 2024; Hewitt et al., 2024; Lu and Cosgun, 2025). These approaches help mitigate the risk of relying solely on a model’s internal memory.

Biomedical workflows are often complex and require calculations, ontology/database queries, and code execution. Otherwise analysis steps are not reproducible and reliable. AI agents can decompose complex workflows into callable steps (calculations, ontology/database queries, code execution) while preserving traces for reproducibility. Open frameworks and systems, such as BioChatter (Lobentanzer et al., 2025) or BioAgents (Mehandru et al., 2025) demonstrate constrained, locally deployable, retrieval-enhanced pipelines for biomedical tasks. Advanced agentic systems for genetics include BioDiscoveryAgent for perturbation-experiment design (Roohani et al., 2025) and a chatbot agent to facilitate family communication of hereditary risk in familial hypercholesterolemia (Walters et al., 2023).

Finally, multi-site collaboration is often required, while raw data cannot be shared. Federated learning enables privacy-preserving training and cross-site collaboration without raw data exchange, aligning with regulatory expectations (Cho et al., 2025; Amin et al., 2024; Calvino et al., 2024; Kolobkov et al., 2024). However, these techniques usually require additional technical expertise.

Taken together, these practices underscore that effectiveness depends not only on model scale but also on task alignment, prompt design, real-time access to knowledge, and auditable reasoning tools, which are key ingredients for trustworthy clinical deployment.

### 4.3 Data and benchmarks

The growing use of generative AI is closely tied to the quality of available datasets and benchmarks. Reliable evaluation and generalization depend not only on model design but also on data diversity, integrity, and task-relevant benchmarking protocols (Nijman et al., 2022; Liu Mingxuan et al., 2023; Chahid et al., 2023; Castro et al., 2020).

LLM applications in genetic diagnostics require reliability; therefore, robust benchmarks are vital for comparing models and ensuring trust. CARDBiomedBench (Bianchi et al., 2025) exemplifies this shift, offering a multi-domain Q&A benchmark in biomedicine. Its design is based on curated expert knowledge and data augmentation, which exposes real gaps in model reasoning and safety, even among state-of-the-art systems. The number of domain benchmarks, reported scores, and proposed tracking methods continues to grow (Labbe et al., 2023; Tarabanis et al., 2024; Ke et al., 2025; Hamilton et al., 2024; Li Shumin et al., 2024; Murphy et al., 2024; O’Sullivan et al., 2024), helping move beyond general-purpose NLP benchmarks toward the nuanced reasoning required in biomedical decision-making.

Recent work in other technical domains has highlighted the threat of benchmark leakage, where models inadvertently see test data during pretraining (Zhou et al., 2025; Ni et al., 2025). Complementing these findings, another study shows a chronological “task contamination” effect: LLMs score markedly higher on datasets released before their training data cutoff than on post-cutoff sets, with supporting evidence from training-data inspection and membership-inference attacks, underscoring how pretraining overlap can inflate zero/few-shot results (Li and Flanigan, 2023). These studies demonstrate how leakage can inflate performance and undermine credibility, motivating leakage-aware protocols and transparent documentation of training data, especially sensitive domains, such as biomedicine.

As noted throughout this review, modern clinical models must integrate diverse data types: text, images, genomics, structured records, which requires both scalable architectures and consistent input quality. Recent methods improve efficiency in multimodal fusion (e.g., contrastive learning, cross-attention) (Golovanevsky et al., 2024; Machado Reyes et al., 2024a; Wu et al., 2024; Kumar Mondol et al., 2024; Zhou et al., 2024; Shirkavand et al., 2023; Machado Reyes et al., 2024b), while preprocessing helps standardize specific modalities (e.g., segmentation (Yuan et al., 2024; Shi et al., 2023; Fu et al., 2024), or facial axes standardization (Alomar et al., 2024)).

Independent of architecture, version pinning (model, tokenizer, prompts, decoding parameters), leakage-aware evaluation, and traceability (logged sources, tool traces, decision checkpoints) improve safety and reproducibility (Raff et al., 2025; Semmelrock et al., 2025). For transparent assessment and regulatory preparedness, healthcare reporting checklists such as MI-CLEAR-LLM are recommended (Park et al., 2024). Together, these developments underscore that the value of LLMs in genetics is not solely defined by model architecture. Equally important are the integrity of training and evaluation datasets, the representativeness of benchmarks, and the methods used to integrate and align multimodal inputs.

### 4.4 Biases

Despite their impressive capabilities, LLMs often reflect biases present in their training data, which can affect clinical utility. Several studies have revealed racial and demographic biases in generated medical reports and other outputs (Yang Yifan et al., 2024; Lin et al., 2024), while others show variations in performance across age-specific manifestations of genetic disorders (Othman et al., 2025) or reviewer experience levels (Levin et al., 2024). Language also remains a critical source of disparity: most biomedical models are English-centric, limiting accessibility and accuracy in other languages. Resources such as MedLexSp for Spanish (Campillos-Llanos, 2023), Chinese medical conversational Q&A corpora (Weng et al., 2023), and domain adaptation efforts for Japanese genetic counseling (Fukushima et al., 2025) demonstrate how localized models and lexicons can help reduce these gaps.

In genomics and precision medicine, the lack of diversity in training data has long limited the generalizability of AI insights for underrepresented groups. Over 80% of genome-wide association studies to date have been conducted on individuals of European ancestry (Schumacher-Schuh et al., 2022), leading to predictive tools that underperform in other populations. For example, polygenic risk scores trained predominantly on Eurocentric cohorts show substantially lower accuracy when applied to individuals of African, Hispanic, or other ancestries, reflecting poor out-of-distribution generalization and exacerbating health disparities (Kidenya and Mboowa, 2024; Step et al., 2024). These gaps highlight that without deliberate interventions to include diverse data, AI systems, including LLMs, may fail to equitably serve marginalized communities.

Overall, recent findings underscore the need for targeted fairness efforts and rigorous ethical evaluation in deploying AI. In practice, we recommend reporting results stratified by site and language (separate performance metrics per hospital/registry and clinical language); notably, even FDA-cleared AI tools rarely report performance by patient demographics, underscoring the importance of transparent subgroup evaluation. Using ancestry-aware sampling during model development (i.e., balancing or weighting cohorts to better reflect the target population) is another key step, alongside technical bias-mitigation measures (Weiner et al., 2025). For instance, integrating data augmentation and algorithmic debiasing techniques can help ensure models maintain consistent performance across subgroups (Jha et al., 2025). We also advice scheduling fairness checks in production, periodic bias audits that monitor performance gaps across demographic subgroups, to catch and remediate any emerging disparities. Such responsible AI practices, combined with language- or population-specific model adaptations, are essential to mitigate bias and promote more equitable clinical AI systems (Weiner et al., 2025).

## 5 Conclusion

As detailed in this review, transformer-based models have made significant progress in various critical tasks within the research and diagnosis of human genetic diseases. Generative AI methods have proven their efficiency in diverse tasks related to knowledge navigation, analysis of clinical and genetic data, and interaction with researchers, medical specialists, and patients. Owing to the peculiar architecture of generative models, they have found their application beyond standard classification tasks, and are now widely used for complex tasks such as genetic variant interpretation, generation of novel biological hypotheses, or prediction of complex epigenomic features for polygenic risk assessment.

Generative AI tools, including LLMs, hold clear potential for supporting various professional roles involved in genetic medicine. For clinical geneticists, LLM-powered systems (described in this article as well as newly developed) can assist in providing definitive diagnosis, prediction of individual risks, and interactions with patients. For researchers and bioinformaticians, such models offer solutions for complicated tasks involving processes of vast amounts of genomic or other high-throughput data. As LLMs mature, we anticipate their deployment in software environments designed to assist these distinct expert groups, enhancing the quality and speed of inherited disease diagnostics.

Naturally, this review cannot cover every tool and model in a field that evolves so rapidly. Rather, it provides a structured overview that can serve as a classifier and guide, helping researchers and practitioners navigate the fast-growing landscape of LLM applications in human medical genomics.

## Data Availability

The original contributions presented in the study are included in the article/Supplementary Material, further inquiries can be directed to the corresponding authors. All code used for data processing, analysis, and figure generation is available at: https://github.com/TohaRhymes/llm_in_diagnostics.
