# Supplementary figures and images for: A systematic review on the generative AI applications in human medical genetics
Source: Front Genet. 2026 Jan 20;16:1694070. doi: 10.3389/fgene.2025.1694070 (PMC12863965; doi:10.3389/fgene.2025.1694070)

# Topic Modeling Scatter (LDA, curated articles)

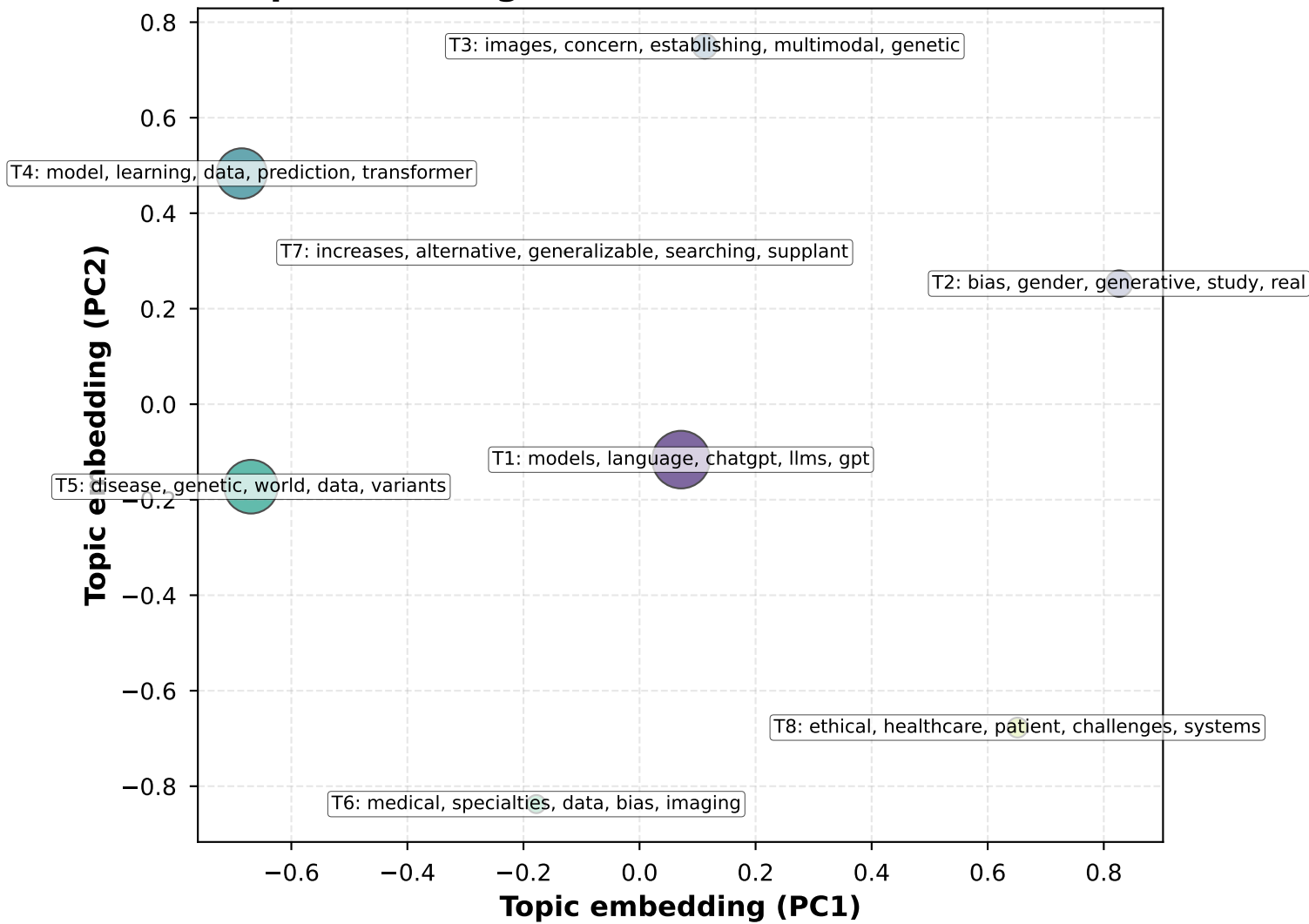

Supplement: Supplementary file 1 [file Image5.pdf]

Source Comparison: Fine-Tuned Analysis (Top-30)  
Context Preserved, Post-hoc Reweighting

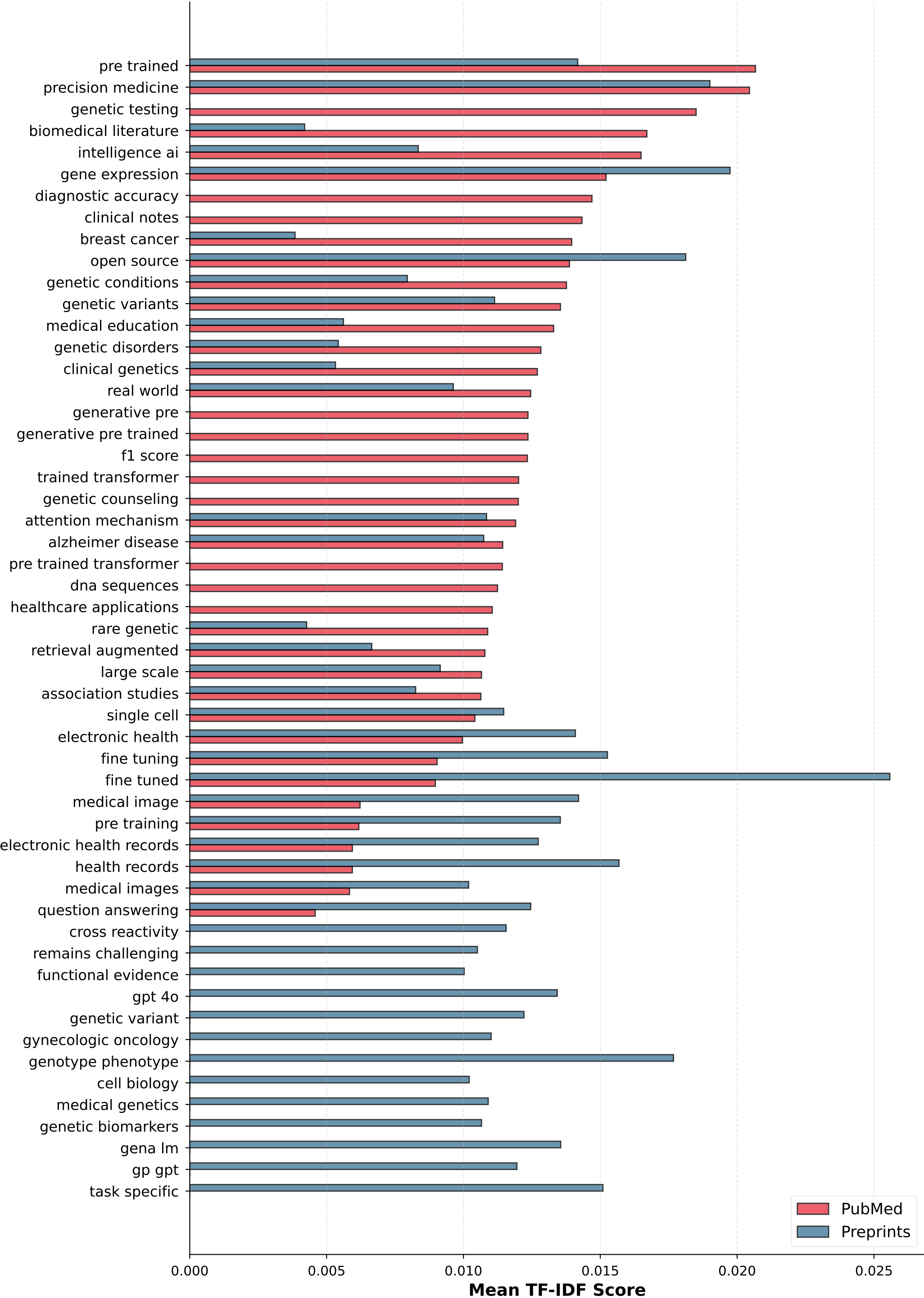

Supplement: Supplementary file 5 [file Image4.pdf]

Source Comparison: Selected Articles (Top-30)  
Before Filtering

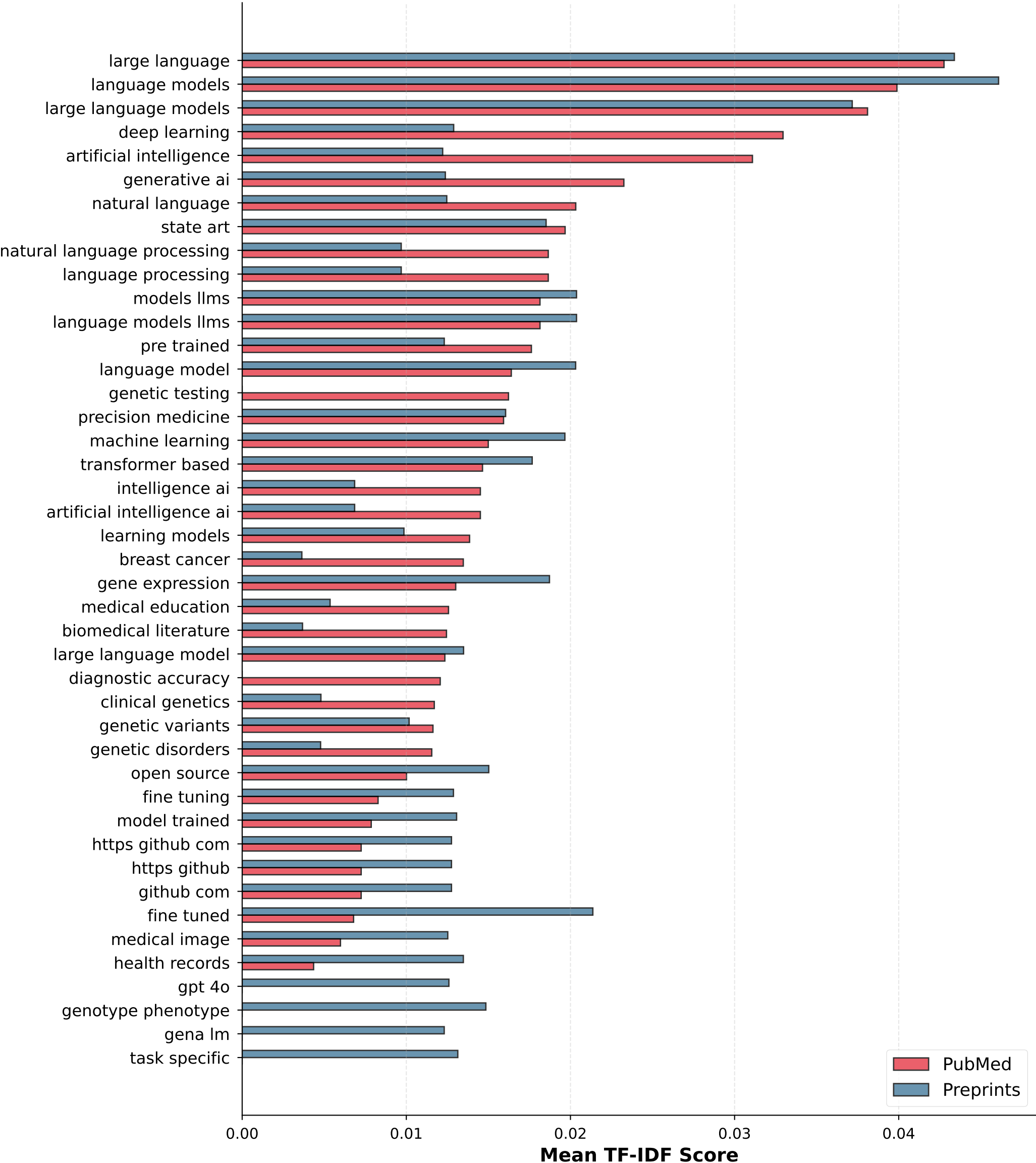

Supplement: Supplementary file 6 [file Image2.pdf]

Source Comparison: Selected Articles + Filtered Phrases (Top-30)

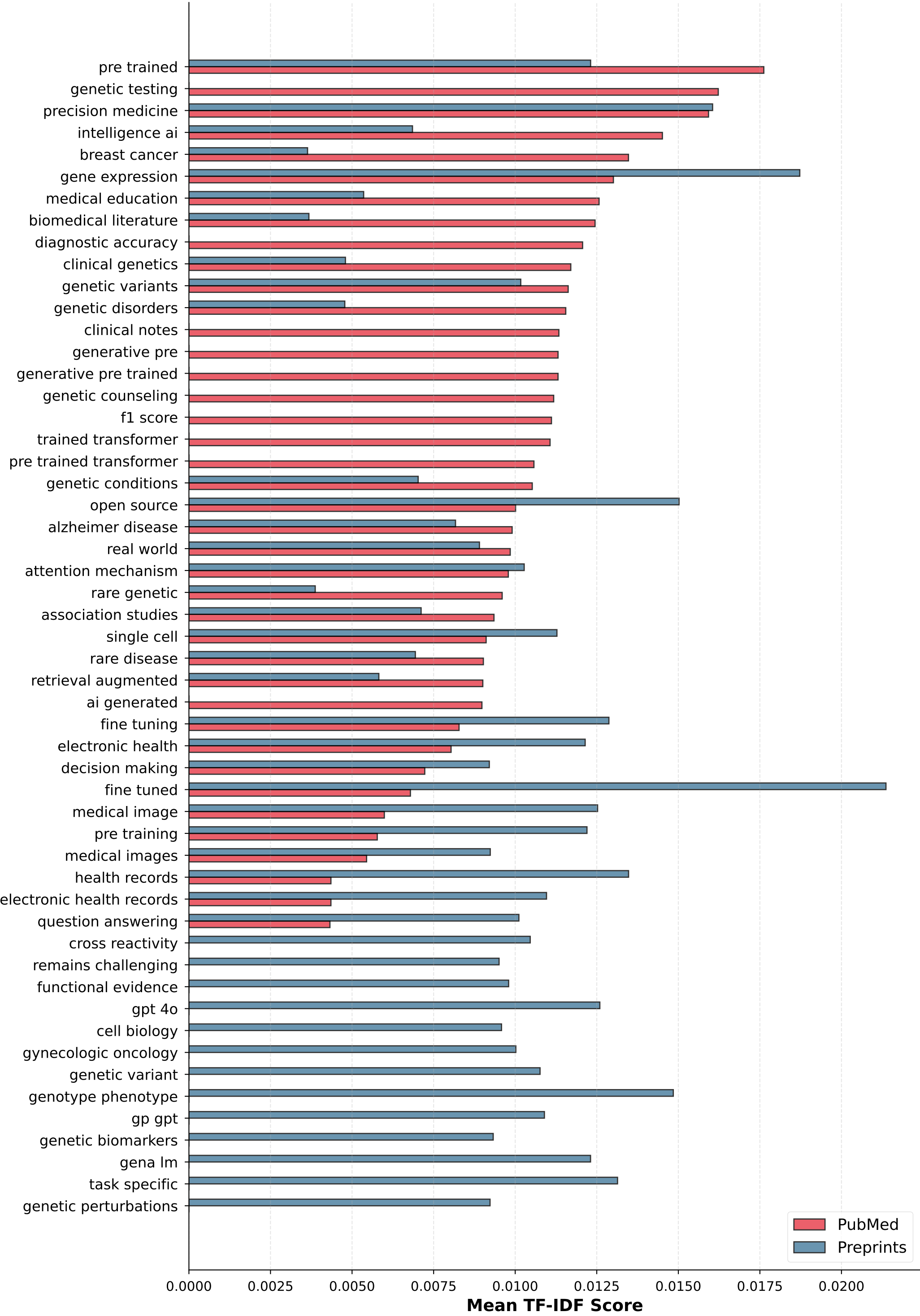

Supplement: Supplementary file 7 [file Image3.pdf]

**A****Full Dataset (Top 30)**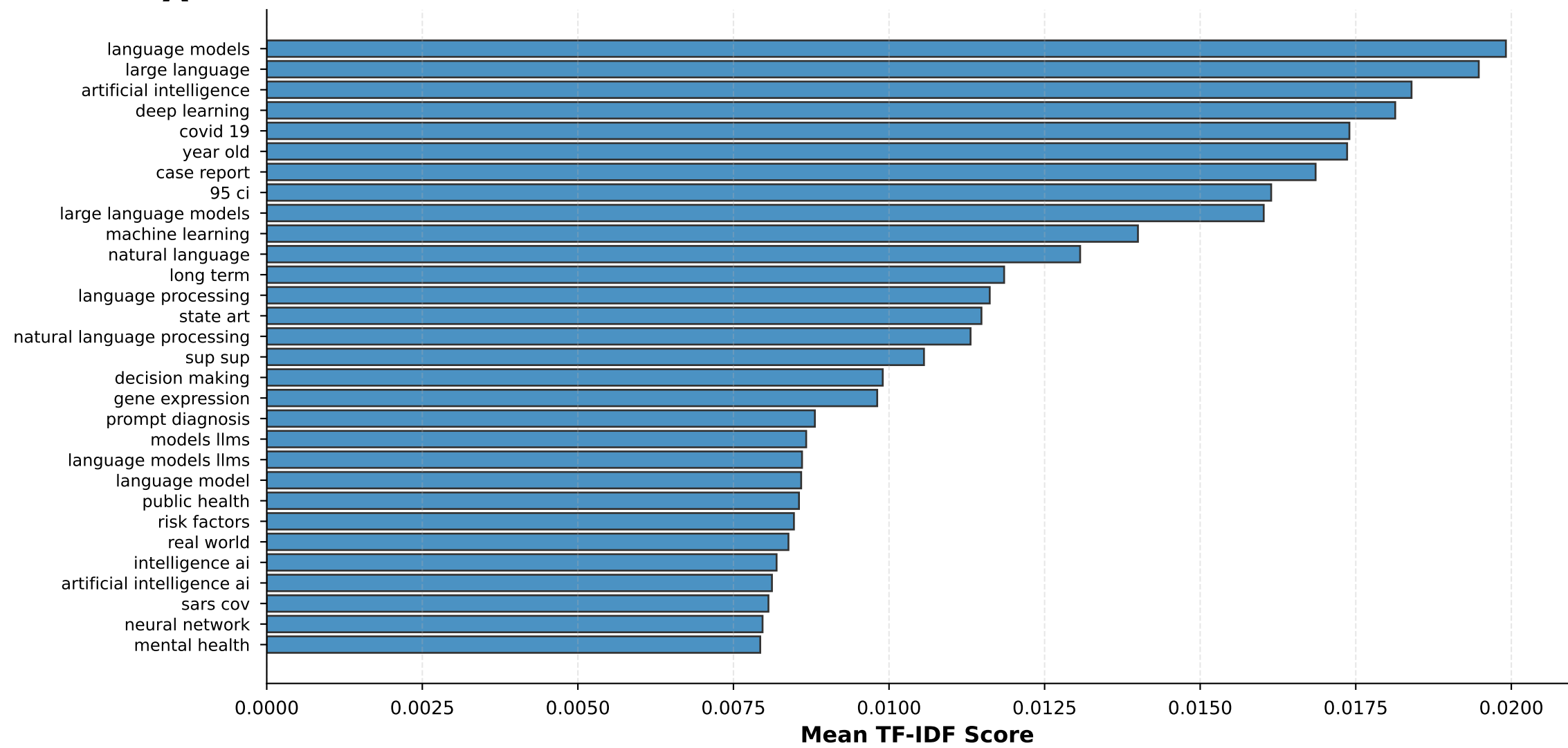**B****Selected Articles (Top 30)**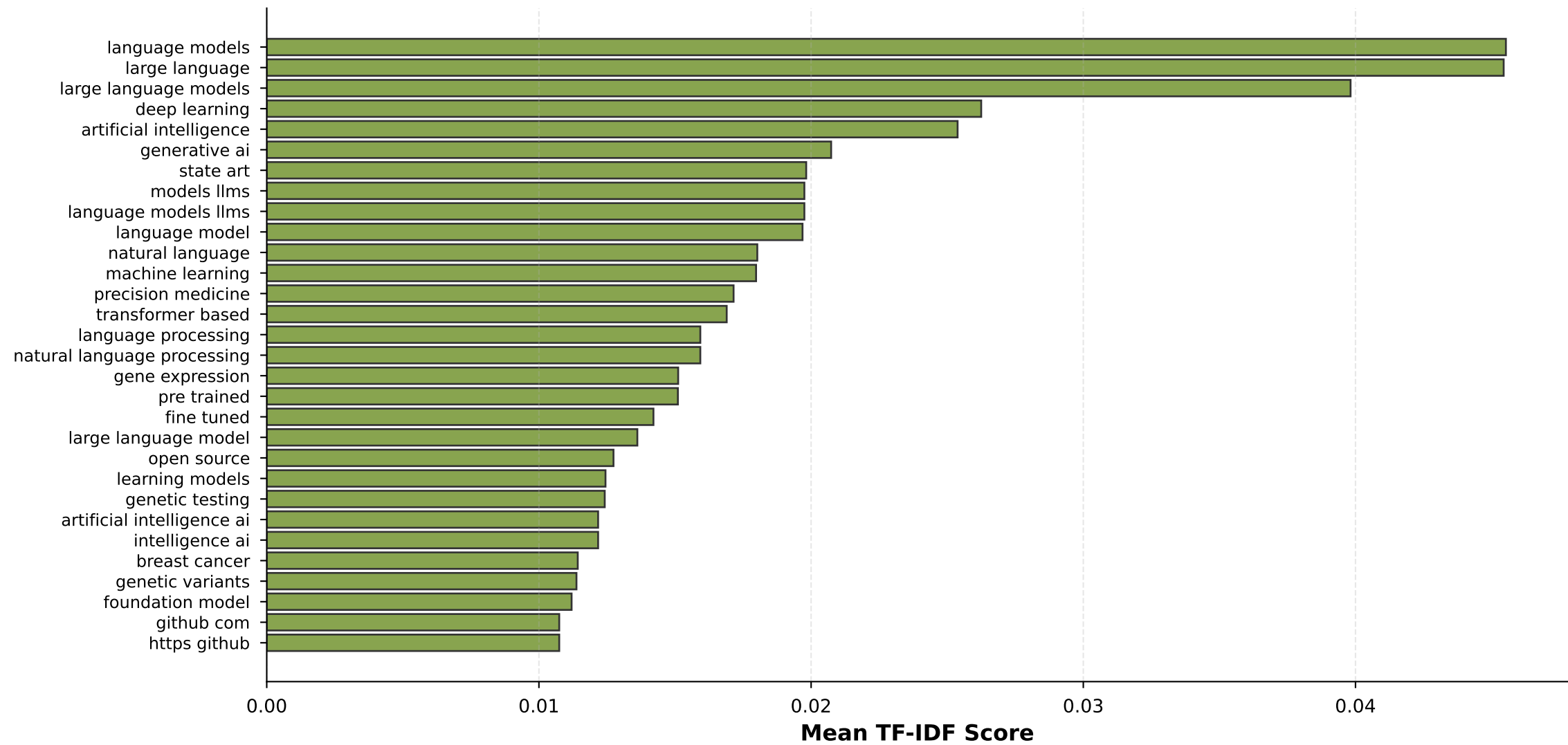**C****Selected Articles + Filtered Phrases (Top 30)**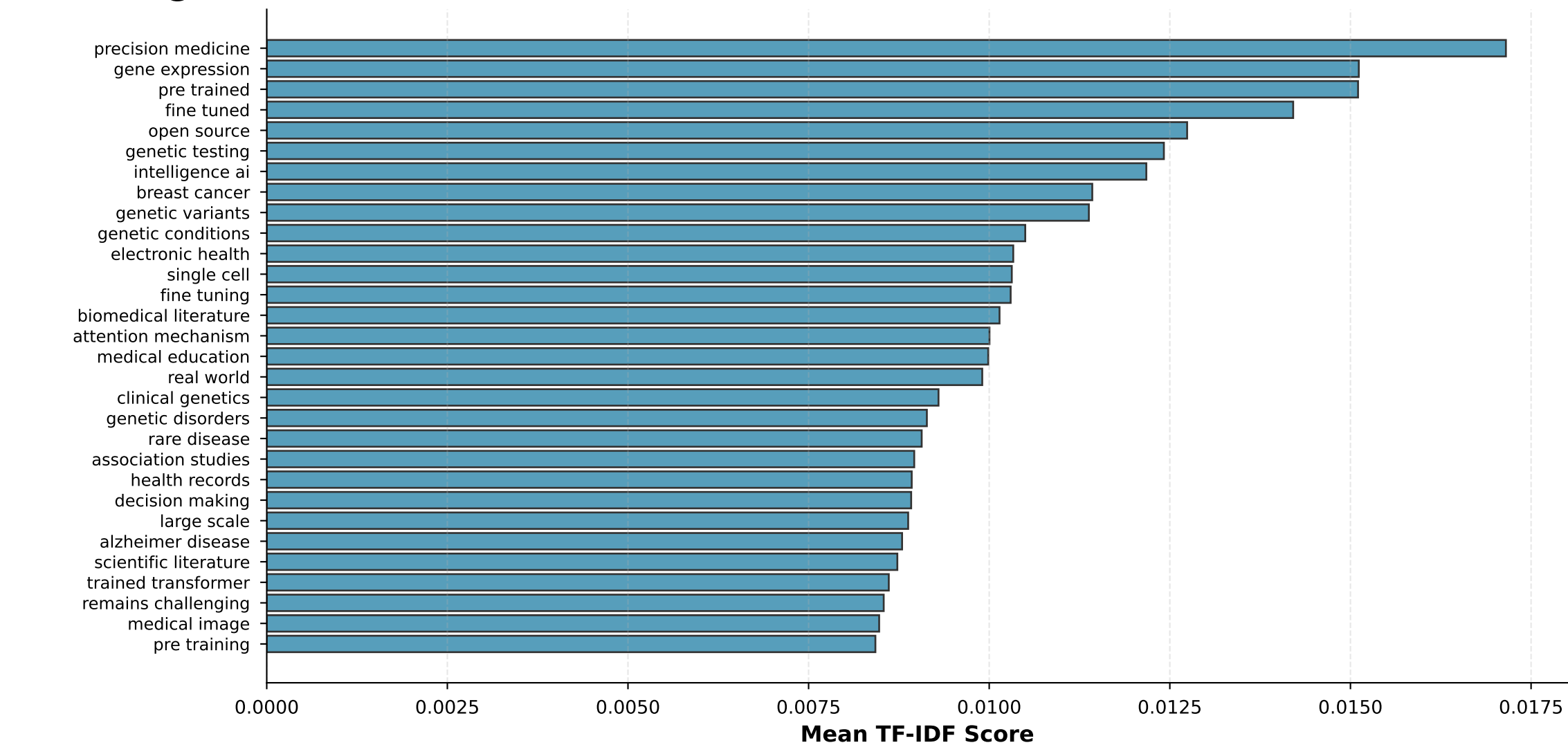

Supplement: Supplementary file 10 [file Image1.pdf]
