# Supplementary material for: A systematic review on the generative AI applications in human medical genetics
Source: Front Genet. 2026 Jan 20;16:1694070. doi: 10.3389/fgene.2025.1694070 (PMC12863965; doi:10.3389/fgene.2025.1694070)
Supplement: Supplementary file 3 [file Presentation1.pdf]

Appendix

Appendix A: Supplementary Tables - Annotated Article Dataset

Two supplementary tables were compiled to support the analysis presented in this study. Supplementary Table 1 (ST1) contains the complete list of articles included after initial collection, deduplication, and manual verification. Supplementary Table 2 (ST2) provides an extended, manually annotated version of the dataset with additional semantic tags and classification columns.

Supplementary Table 1 (ST1) presents the cleaned dataset after the removal of duplicates and initial triage. Duplicate entries were identified not only through automatic preprocessing but also through joint manual assessment by two researchers, ensuring a consistent and conservative approach to inclusion. ST1 includes metadata such as the article title, abstract, source, review status, and initial relevance tag.

Supplementary Table 1 (ST2) expands upon this initial dataset by including additional annotations used in the systematic analysis. These include fine-grained labels for specific tasks inside these stages (`final_category`, `subcategory`), and three binary relevance flags (`not_relevant`, `partly_relevant`, `relevant`). 27 manually selected articles were also added at this stage (eight highly relevant and 19 partially relevant), resulting in a total of 325 articles in ST2. These additions were motivated by expert review and targeted searches within the originally collected corpus and cited references.

Detailed descriptions of column meanings and classification codes are available in the project GitHub repository (https://github.com/TohaRhymes/llm_in_diagnostics)

Appendix B: F-IDF and Filtering Methods

To characterize the semantic landscape of LLM applications in medical genomics, we employed two complementary text mining approaches: Term Frequency-Inverse Document Frequency (TF-IDF) analysis for identifying domain-specific terminology, and Latent Dirichlet Allocation (LDA) for discovering latent thematic structure. Both methods were applied to article titles and abstracts from the curated dataset.

Term Frequency-Inverse Document Frequency (TF-IDF) Analysis

As mentioned earlier, TF-IDF helped to identify areas of the research in applications of LLMs. It was applied at multiple stages: the full corpus (51613 records after deduplication), the curated review set (195 articles), and filtered variants where generic AI/ML phrases were removed (to move beyond obvious LLM keywords such as "language model", "deep learning" -- see full pattern list in the source code).

Bigram-trigram TF-IDF scores were computed (scikit-learn's `TfidfVectorizer` with `ngram_range=(2,3)` and `max_features=1000`), lower-casing and removing English stop-words plus custom artifacts (e.g., "et al").
Additionally, a context-preserving fine-tuning approach was implemented. This method first trains the TF-IDF model on the curated dataset (195 articles) with full terminology context, then applies post-hoc reweighting by zeroing out generic AI/ML anchor terms and renormalizing the document vectors. This preserves the semantic context during initial feature extraction while down-weighting generic phrases in the final ranking. The fine-tuned analysis confirms that the domain-specific trends reported in the main text (e.g., precision medicine, gene expression, genetic testing) remain stable once obvious anchors are de-emphasized, demonstrating that our findings are robust to different filtering strategies.

Sources within the curated set were compared by stratifying PubMed (n=131) versus preprints (bioRxiv/medRxiv/arXiv; n=64). For each comparison, the union of the two top-30 lists was applied. This helped to capture the shift from generic to domain-specific terminology and highlight complementary emphases between peer-reviewed and preprint venues.

Additional representations of TF-IDF analysis are shown in four supplementary figures: Supplementary Figure 1 (three-stage progression of TF-IDF after selecting articles and filtering words), Supplementary Figure 2 (comparison of sources before filtering), Supplementary Figure 3 (comparison of sources after filtering), and Supplementary Figure 4 (comparison of sources using fine-tuned analysis).

In addition to standard English stop-words, generic AI/ML phrases and artifacts were excluded to surface domain-specific terminology. The final list included the following common terms: large language, language model, llm, llms, generative ai, foundation model, foundation models, deep learning, deep neural, neural network, neural networks, machine learning, artificial intelligence, artificial neural, natural language, language processing, nlp, transformer model, transformer models, reinforcement learning, supervised learning, unsupervised learning, state art, based, using, https, github, model, models, learning, data.

Supplementary Figure 1
Progression of TF-IDF analysis from full corpus to filtered insights. (A) Full dataset (51,613 articles): generic anchors dominate; (B) Selected articles (195): core themes retained; (C) Selected articles + Filtered words: domain-specific trends (e.g., precision medicine, gene expression, human phenotype ontology, single cell) become prominent. Horizontal bars show the top-30 phrases per stage.

Supplementary Figure 2
Source comparison before filtering generic phrases. Grouped bars show TF-IDF scores on the same scale for the union of top-30 phrases across PubMed (n=128) and preprints (n=64). Strong overlap (57%) indicates consensus on core topics before filtering.

Supplementary Figure 3

Source comparison of research trends after article selection and filtering of generic AI/ML phrases. Grouped bars show actual TF-IDF scores for the union of top-30 phrases from PubMed (n=128) and preprints (n=64) on the same scale. Overlap drops to 23%, revealing distinct emphases: PubMed emphasizes clinical/translational terms while preprints highlight computational methods.

Supplementary Figure 4
Source comparison using fine-tuned analysis (context preserved, post-hoc reweighting). This analysis includes training TF-IDF with full context, then down-weighting generic AI/ML terms. Results confirm that domain-specific trends remain stable (27% overlap between sources), validating the filtered analysis approach. The fine-tuned method preserves semantic relationships while surfacing specific research emphases.

Latent Dirichlet Allocation (LDA) Topic Modeling

To address the visual representation of topic overlap, we performed Latent Dirichlet Allocation (LDA) topic modeling on the curated dataset. Eight topics were extracted using gensim with automatic hyperparameter optimization. Topic similarity was quantified using Jensen-Shannon divergence between topic-word distributions, and topics were arranged in 2D space using a custom force-directed layout algorithm that positions similar topics closer together. The resulting scatter plot (Supplementary Figure 5) displays topics as bubbles sized by prevalence, with labels showing the top five terms per topic. This visualization provides an intuitive view of how topics relate to each other and their relative importance in the literature.

Supplementary Figure 5.
Topic modeling visualization showing overlap and relationships between themes. Eight LDA topics fitted on the curated dataset (n=195) are displayed in 2D space using ensen-Shannon divergence-based layout. Bubble size reflects topic prevalence. Labels show top five terms per topic. Topics closer together share more vocabulary, illustrating the semantic landscape of LLM applications in medical genomics.

Supplementary Table 3 (ST3) presents topic modeling metadata from LDA analysis, including topic identifiers, visualization coordinates, prevalence values, and the top five representative terms for each of eight identified topics.

Supplementary Table 4 (ST4) provides the document-topic probability distribution matrix, where each row represents one article from ST2 and columns represent the probability of assignment to each of the eight topics, plus source categorization.
